# Retinal Ganglion Cells Die by Necroptotic Mechanisms in a Site-Specific Manner in a Rat Blunt Ocular Injury Model

**DOI:** 10.3390/cells8121517

**Published:** 2019-11-26

**Authors:** Chloe N. Thomas, Adam M. Thompson, Zubair Ahmed, Richard J. Blanch

**Affiliations:** 1 Neuroscience and Ophthalmology, Institute of Inflammation and Ageing, College of Medical and Dental Sciences, University of Birmingham, Birmingham B15 2TT, UK; c.thomas.4@bham.ac.uk (C.N.T.); a.thompson@exeter.ac.uk (A.M.T.); 2 School of Biomedical Sciences, Institute of Clinical Sciences, College of Medical and Dental Sciences, University of Birmingham, Birmingham B15 2TT, UK; 3 Academic Department of Military Surgery and Trauma, Royal Centre for Defence Medicine, Birmingham B15 2TT, UK; 4 Department of Ophthalmology, University Hospitals Birmingham NHS Foundation Trust, Birmingham B15 2TT, UK

**Keywords:** necroptosis, retinal ganglion cells, cell death, trauma, traumatic optic neuropathy, commotio retinae, photoreceptors

## Abstract

Closed-globe injury can cause visual loss in military and civilian populations, with retinal cell death, including retinal ganglion cell (RGC) degeneration, leading to irreversible blindness. RGC and optic nerve (ON) degeneration after eye or head injury is termed traumatic optic neuropathy (TON). There are currently no treatments for RGC loss, therefore novel therapeutics to prevent RGC death or promote axonal regeneration are a priority. We investigated necroptotic signaling mechanisms in a rat blunt ocular injury model. After bilateral blunt trauma, protein expression and retinal localization of necroptosis pathway members (receptor interacting protein kinase 1, RIPK1; receptor interacting protein kinase 3, RIPK3; and mixed lineage kinase domain like pseudokinase, MLKL) were assessed by Western blot and immunohistochemistry (IHC), and potent necroptosis inhibitor Necrostatin-1s (Nec-1s) was delivered by intravitreal injection to one eye and vehicle to the contralateral eye. RGC and photoreceptor survival were assessed by cell counting and outer nuclear layer (ONL) thickness measurements on histology. The neuroprotective effects of Nec-1s were assessed in primary retinal culture by βIII-tubulin^+^ RGC cell counts. MLKL protein expression were upregulated at 48 h after injury and MLKL immunolocalised to retinal binding protein with multiple splice (RBPMS)^+^ RGC, inner nuclear cells and ONL cells, specifically at the retinal injury site. RIPK3 expression did not increase but RIPK3 co-immunolocalised with RBPMS^+^ RGC in intact and injured retinae. In vitro, a Nec-1s concentration of 0.01 pg/µL was RGC neuroprotective. In the blunt ocular injury rat model, Nec-1s prevented RGC death at the center of the impact site but did not protect against ONL thinning or provide functional restitution. RGC degeneration in our blunt ocular injury model is site-specific, with necroptosis driving death at the center of the focal impact site.

## 1. Introduction

Ocular injuries are common, occurring in 10% of all military casualties [1] and have a lifetime prevalence of 20% in the civilian population [2]. The light-sensitive membrane at the posterior portion of the eye, the retina, contains many cells including photoreceptors and retinal ganglion cells (RGC), which convert light into electrical signals and relay this information to the brain for processing. RGC exist in the ganglion cell layer (GCL) and their axons form the brain connection from the eye to the brain, the optic nerve (ON). The retina is an extension of the central nervous system (CNS), which means that if retinal cells, including photoreceptors and RGC, are injured and lost through disease or injury, they are not endogenously replaced. Thus, their preservation is important to prevent irreversible loss of vision.

Traumatic optic neuropathy (TON) can be caused by head or eye injury and is associated with RGC and axonal degeneration, causing irreversible blindness [3,4]. The ON can be injured either directly (e.g., penetrating ocular injury, bony fragment damage with optic canal, and ON sheath hematomas) or indirectly after traumatic head or eye injury (e.g., blunt eye injury and blast) [5,6,7]. Blunt ocular injury causes injury to RGC and photoreceptors (commotio retinae) [8]. There are currently no effective treatments for TON [9], despite TON occurring with an annual incidence of at least 1/1,000,000 in the civilian population [10], and in up to 20% of military eye injuries [11]. TON can be studied using animal models replicating blunt and blast ocular injuries [12,13,14,15] and ON crush (ONC) [16,17,18].

Necroptosis (controlled necrosis) is a regulated cell death pathway resulting in cell leakage, organelle swelling, cytoplasmic granulation and the initiation of an inflammatory response [19,20]. Receptor-interacting protein kinase 1 (RIPK1) and 3 (RIPK3) are essential in tumor necrosis factor (TNF)-induced necroptosis. After TNF stimulation at the TNF receptor, complex I is formed, which involves polyubiquitinated RIPK1 and activation of the pro-survival NF-κβ pathway [21]. Reduced pro-survival signals lead to complex I dissociation and the formation of complex IIa, which normally causes apoptosis. However, if caspase-8 activation is inhibited, either through endogenous or pharmacological inhibitors, then RIPK1 and RIPK3 associate alongside mixed lineage kinase domain-like (MLKL) and form complex IIb. RIPK3 phosphorylates MLKL at Ser345 [22], inducing its translocation to the plasma membrane, triggering membrane permeabilization and necroptotic death [23].

Necroptosis promotes neuronal cell death in trauma, neurodegeneration and eye disease [24]. RIPK1 expression is elevated in a rat intracerebral hemorrhage model and its inhibition or genetic knockdown reduces brain injury severity [25]. Further, in a mouse model of Alzheimer’s disease (APP/PS1 mice) treatment with necroptosis inhibitor Necrostatin-1 (Nec-1) reduces cognitive impairments, β-amyloid and tau abnormalities [26]. Nec-1 also improves histopathology and function in models of traumatic brain and spinal cord injury [27,28]. In a retinal ischemia-reperfusion model, there is increased expression of RIPK1 and RIPK3 and inactive caspase-8 present in the GCL and Nec-1 administration reduced retinal damage and provided RGC neuroprotection in vivo and in vitro [29,30]. However, Nec-1 has some off-target effects, as it also inhibits indoleamine-2,3-dioxygenase (IDO), which has a role in immunomodulatory function [30]. Therefore, in this study we used an alternative more stable analogue called Necrostatin-1 stable (Nec-1s), which has higher affinity and specificity than Nec-1, is >1000 fold more selective for RIPK1 than other human kinases and does not interfere with IDO [31,32].

Previously, we have shown that RGC die by caspase-2-dependent mechanisms in the peripheral portion of the focal retinal impact site, but we did not identify the mechanisms of RGC death at the center of the impact site, where there is the greatest RGC death [33]. Blanch et al., have also demonstrated that caspase-9 promotes photoreceptor death and a lack of caspase-8 activation after blunt ocular injury, implying that necroptotic mechanisms could have been activated in this model [34]. In this current study, we have demonstrated necroptotic death occurring in the retina at early time points in a blunt ocular injury rat model and inhibition of necroptosis by Nec-1s prevents some RGC death at the center of the impact site but did not provide functional RGC protection.

## 2. Materials and Methods

### 2.1. Experimental Design

**Figure 1:**
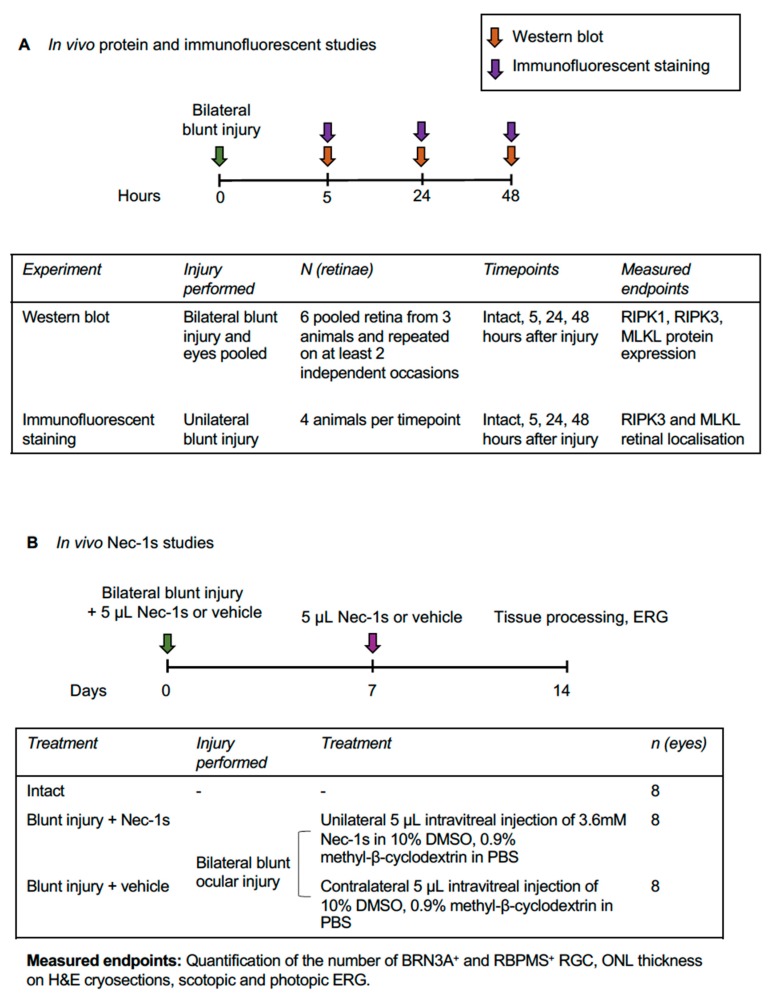
Experimental design. (**A**) In vivo protein and immunofluorescent staining studies; (**B**) In vivo Nec-1s study. Abbreviations: DMSO = Dimethyl Sulfoxide; ERG = electroretinogram; MLKL = mixed lineage kinase domain-like; Nec-1s = Cecrostatin-1s; PBS = phosphate buffered saline; RIPK1 = receptor interacting protein kinase 1; RIPK3 = receptor interacting protein kinase 3.

To determine the role of necroptosis in RGC and photoreceptor death after blunt ocular injury, groups of adult female adult Lister Hooded rats were subjected to unilateral or bilateral blunt ocular injury under anesthesia, as previously described [15]. Western blot and immunohistochemistry were performed at 5, 24 and 48 h after injury to determine RIPK1, RIPK3 and MLKL retinal protein levels and cellular localization (Figure 1A). Nec-1s, a selective necroptosis inhibitor, was used to manipulate RGC survival in primary retinal cultures in vitro and in a rat blunt ocular injury model in vivo (Figure 1B). RGC and photoreceptor functional and structural assessments were performed using scotopic and photopic electroretinogram (ERG) recordings, and the number of retinal binding protein with multiple splicing (RBPMS)^+^ and brain-specific homeobox/POU domain protein 3A (BRN3A)^+^ RGC counted, and outer nuclear layer (ONL) thickness measured in retinal sections.

### 2.2. Animal Care and Procedures

Animal procedures were licensed by the UK Home Office, approved by the University of Birmingham’s Animal Welfare and Ethical Review Committees and conducted in accordance with the ARVO Statement for the Use of Animals in Ophthalmic and Vision Research. Female Lister-hooded rats, weighing 180–220 g, were purchased from Charles River Laboratories (Margate, UK), kept on a 12 h light–dark cycle with a daytime luminance of 80 lux and fed and watered ad libitum. Surgery and ERG recordings were performed under inhalational anesthesia with 2–3% isoflurane in oxygen. Blunt ocular injury was induced as previously described [15]. Briefly, a 0.095 g spherical plastic pellet was fired using compressed air to directly impact the inferior scleral surface at a speed of 20 m/s. This creates a localized retinal injury-causing photoreceptor and RGC degeneration by 14 days post injury (dpi) and extensive loss of retinal function, with ERG amplitude reduced to <50% of intact values [15].

### 2.3. Western Blot

Rats were killed at 5, 24 and 48 h after bilateral blunt ocular injury, as well as uninjured intact controls, by overdose of anesthetic (six pooled retinas from three animals receiving bilateral blunt injury and repeated on at least two independent occasions). Protein extraction and Western blot were performed as previously described [35]. Briefly, retinal proteins were extracted in ice-cold lysis buffer supplemented with protease inhibitor cocktail (Sigma, Poole, UK). Protein extracts were denatured by heating to 90 °C for 5 min and 40 µg of protein/lane was separated on 12% Tris-glycine sodium dodecyl sulphate (SDS) gels, transferred to polyvinylidene fluoride (PVDF) membranes (Millipore, Watford, UK), probed with appropriate primary and secondary antibodies (Table 1) and specific protein bands were detected using chemiluminescence by exposure to photographic film (GE Healthcare, Little Chalfont, UK). Blots were repeated on at least two independent occasions. Integrated band intensity was measured using the built-in gel analysis macros in Fiji Image J [36] and displayed as % of loading control (β-actin). Error bars represent standard error of the mean (SEM).

### 2.4. Intravitreal Injection of Nec-1s

Nec-1s (Bio Vision Incorporated, CA, USA) was reconstituted at 3.6 mM in 10% dimethyl sulfoxide (DMSO) and 0.9% methyl-β-cyclodextrin (Sigma) in sterile phosphate buffered saline (PBS). Bilateral blunt ocular injury was performed and 5 µL of Nec-1s administered by unilateral intravitreal injection and 5 µL of vehicle (10% DMSO, 0.9% methyl-β-cyclodextrin in PBS) was injected into the contralateral eye. Injections were performed immediately after injury and at 7 dpi until culling and tissue collection at 14 dpi (n = eight eyes/treatment, eight animals total). Uninjured animals were culled without intravitreal injections as intact controls (n = eight animals). Animals were intracardially perfused under terminal anesthesia and eyes processed for immunohistochemistry.

### 2.5. Tissue Preparation for Immunohistochemistry

Tissues were prepared for immunohistochemistry as previously described [33]. Briefly, at 5 and 48 h after unilateral blunt injury (n = four rats per experimental group), rats were killed by overdose of anesthetic and intracardially perfused with 4% paraformaldehyde (PFA) in PBS. The anterior segment was removed and small cuts to identify the injury site were made prior to cryoprotection in a graded series of sucrose solution. Tissues were embedded in optimal cutting temperature compound (OCTc), sectioned in a plane parallel to that running between the injury site and the optic disc at a thickness of 15 µm using a cryostat (Brights Instruments, Huntingdon, UK) and adhered onto SuperFrost^TM^ (Fisher Scientific, Loughborough, UK) coated glass microscope slides, and stored at −20 °C until required.

### 2.6. Immunohistochemistry Protocol

Frozen sections were left to thaw for 20 min and washed 3 × 5 min in PBS, followed by 20 min permeabilization and blocking non-specific sites in PBS containing 1% Triton-X-100 (Sigma) and 3% bovine serum albumin (BSA; Sigma). Sections were incubated overnight at 4 °C with appropriate primary antibodies (Table 1) in 0.5% Tween-20 and 3% BSA, before washing 3 × 5 min in PBS and incubating with relevant secondary antibodies (Table 1) at room temperature (RT). Sections were washed 3 × 5 min in PBS and then mounted in Vectashield mounting medium containing 4′,6-diamidino-2-phenylindole (DAPI) (Vector Laboratories, Peterborough, UK). Controls with omitted primary antibody were included in each run and these were used to set the background threshold levels prior to image capture. Sections were viewed under an AxioPlan 2 epi-fluorescent microscope equipped with an AxioCam HRc, controlled using Axiovision Software (all from Zeiss, Hertfordshire, UK). Images were captured and analyzed by an investigator masked to the treatment conditions.

### 2.7. Electroretinography (ERG)

ERG were recorded (HMsERG—Ocuscience, Kansas City, MO, USA) at 7 and 14 dpi and in uninjured controls and were interpreted using ERG View (Ocuscience). Animals were dark-adapted overnight and prepared for ERG under dim red light (>630 nm). Scotopic (dark-adapted) flash ERG were recorded from −2.5 to +1 log units with respect to standard flash in half log unit steps and photopic (light-adapted) flash ERG were recorded with background illumination of 30,000 mcd/m^2^ over the same range. DTL fiber (Unimed Electrode Supplies, Farnham, UK) corneal electrodes with pressure-molded Aclar (Agar scientific, Stansted, UK) contact lenses were used with needle skin electrodes (Unimed). ERG traces were analyzed using the manufacturer’s semi-automated software ERG View (Ocuscience) and marker position manually verified and adjusted where necessary by a masked observer. Photopic negative response (PhNR) was recorded as a measure of RGC function.

### 2.8. Assessment of ONL Thickness

Animals were culled at 14 dpi and eyes processed as for immunohistochemistry. ONL thickness was measured by a masked observer, as previously described, to determine photoreceptor survival [34]. Briefly, seven retinal sagittal sections per eye, stained with Hematoxylin and Eosin (H&E), were analyzed (to account for variability with respect to distance from the impact site) through the optic nerve head (ONH) and center of the impact site (0 μm) and at 600, 1200 and 1800 μm either side of this plane. Sections were scanned using a Zeiss AxioScan Z1 slide scanner and the ONL manually segmented in Adobe Photoshop by a masked observer, and the ONL area was divided by the length of the retinal segment to give an average ONL thickness in pixels.

### 2.9. Assessment of RGC Survival

RGC survival was quantified using immunohistochemistry for BRN3A [37,38] and RBPMS [39,40]. The number of BRN3A^+^ and RBPMS^+^ immunostained RGC were counted by a masked observer, in the GCL across the entire retinal section. Seven retinal sagittal sections per eye were counted: at the optic disc and center of the impact site (0 μm), and at 600, 1200 and 1800 μm to either side of this plane, and quantified as the number of BRN3A^+^ or RBPMS^+^ RGC per mm of retina.

### 2.10. Primary RGC Culture and Immunocytochemistry

Adult 6–8 week-old Sprague Dawley rats (170–220 g; Charles River) were killed by overdose of anesthetics and retinae dissected and dissociated into single cells with a Papain dissociation kit (Worthington Biochem, NJ, USA) [35]. A density of 125,000 retinal cells, containing enriched populations of RGC, were plated per well in 8-well chamber slides (BD Biosciences) pre-coated with 100 μg/mL poly-D-lysine and 10 μg/mL laminin. Cells were incubated in 300 μL of supplemented Neurobasal-A (Invitrogen) containing 2% *v*/*v* B27 supplement (Life Technologies, Invitrogen), 0.5 mM L-glutamine (Invitrogen) and 0.5% *v*/*v* Gentamycin (50 mg/mL Invitrogen), at 37 °C in 95% O_2_ and 5% CO_2_. After 1-day incubation, 200 μL of supplemented media containing various concentrations of Nec-1s was added to the wells, to make a final concentration of 0.01–100 pg/μL of Nec-1s in 500 μL of media. Cells were then fixed with 4% PFA in PBS, blocked in 0.1% Triton-X-100, incubated in primary antibody solution (Table 1) in antibody diluting buffer (0.5% Tween-20, 3% BSA in PBS) followed by incubation with secondary antibody both for 1 h at RT. Slides were washed in PBS mounted with Vectashield mounting media containing DAPI (Vector Laboratories Ltd.), and imaged using the AxioPlan 2 epi-fluorescent microscope (Carl Zeiss). RGC were stained using βIII-tubulin, a neuronal marker which labels all retinal neurons in the mixed culture (bipolar cells, interneurons), and not specifically RGC [41,42,43], and 45 images per well randomly imaged, and βIII-tubulin^+^ RGC (small round cells with a DAPI stained nucleus and green βIII-tubulin staining in the cytoplasm and have one axon) counted and displayed as average number of βIII-tubulin^+^ RGC per mm^2^. Primary retinal cultures were performed in duplicate and repeated on three independent occasions.

### 2.11. Statistics

Power calculations performed in G*Power (v. 3.1.4, Kiel University, Germany) indicated for Western blotting, n = three animals per time point had 82% power to detect a 1-fold change in protein levels, (assuming standard deviation = 20% band intensity); for ERG assessment (more variable than structural measures, therefore less powerful) eight animals had a power of 88% to detect a moderate (f = 0.25) treatment effect (correlation among repeated measures = 0.5 from past data) [15].

Statistical analysis was carried out using SPSS (IBM Corp, Armonk, NY, USA) and GraphPad Prism version 7.00. (GraphPad Software, La Jolla California). The data were tested for normality using the Shapiro–Wilk test. Western blot data were analyzed using one-way analysis of variance (ANOVA) and post-hoc Tukey tests with p values corrected for multiple comparisons. In experiments where multiple comparisons were made, the p values were corrected with Bonferroni correction and the correction factor is stated. ERG amplitudes, BRN3A^+^ and RBPMS^+^ RGC count data and ONL thicknesses were analyzed using generalized estimating equations (autoregressive correlation matrix, gamma distribution with log link). Data are reported as mean ± standard error of the mean (SEM).

## 3. Results

### 3.1. MLKL Protein Expression was Higher 5 h after Blunt Ocular Injury Compared with Intact Eyes, but RIPK1 and RIPK3 Expression Did Not Change

**Figure 2:**
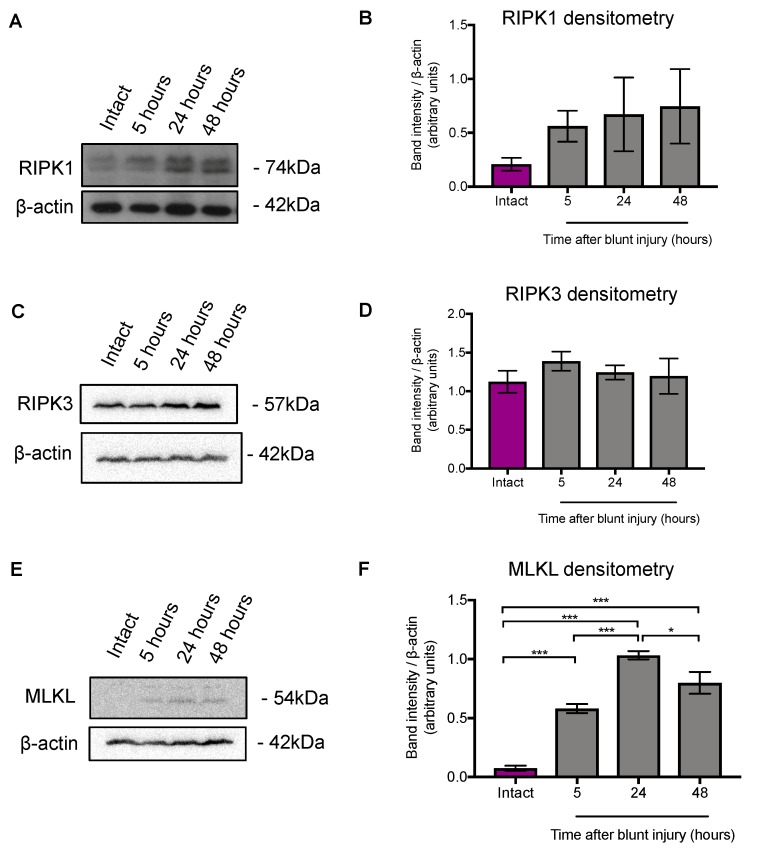
Receptor interacting protein kinase 1 (RIPK1), receptor interacting protein kinase 3 (RIPK3) and Mixed Lineage Kinase Domain like Pseudokinase (MLKL) protein expression after blunt ocular injury. Representative Westerns blot with β-actin as a loading control. (**A**,**B**) RIPK1 showed no changes in in protein expression after blunt ocular injury (*p* = 0.494, ANOVA). (**C**,**D**) RIPK3 protein expression remained constant after injury (*p* = 0.677, ANOVA). (**E**,**F**) MLKL protein expression significantly increased at 5, 24 and 48 h after blunt ocular injury (*p* < 0.0001, ANOVA, post-hoc Tukey *** *p* < 0.0001, * *p* < 0.05). Blots represent six pooled retina, from three animals receiving bilateral blunt injury and repeated on at least two independent occasions. Error bars represent mean ± SEM.

We first determined if the expression of key necroptotic proteins, RIPK1 and RIPK3 as well as downstream MLKL, were altered in our blunt ocular trauma model by performing bilateral blunt ocular injury collecting retinal tissue for immunofluorescent staining and Western blot at 5, 24 and 48 h after injury, as well as from separate intact control animals. Protein expression of RIPK1, RIPK3, MLKL were investigated using Western blot (n = three animals per experimental group, six pooled retinae, repeated on at least two independent occasions). Representative blots are shown in Figure 2A,C,E, and densitometry of band intensities in Figure 2B,D,F.

There was strong evidence of an increase in retinal MLKL expression at 5, 24 and 48 h after blunt ocular injury (*p* < 0.0001, ANOVA; Figure 2E,F), with a band intensity compared to β-actin peaking at 1.033 ± 0.035 24 h after injury (13.8-fold increase from intact; Tukey’s post hoc *t*-test *p* < 0.0001). We observed that the levels of RIPK1 in the retina increased at 5, 24 and 48 h after blunt ocular injury compared to levels in intact controls by Western blot (Figure 2A) but subsequent densitometry (Figure 2B) showed no changes (*p* = 0.494, ANOVA). The levels of RIPK3 remained unchanged (*p* = 0.677, ANOVA; Figure 2C,D).

These results show that MLKL was significantly upregulated in the retina after blunt ocular trauma, which is a key step in necroptosis.

### 3.2. RIPK3 is Localized to BRN3A^+^ RGC whilst MLKL is Localized to OPL, ONL, INL and RBPMS^+^ RGC

**Figure 3:**
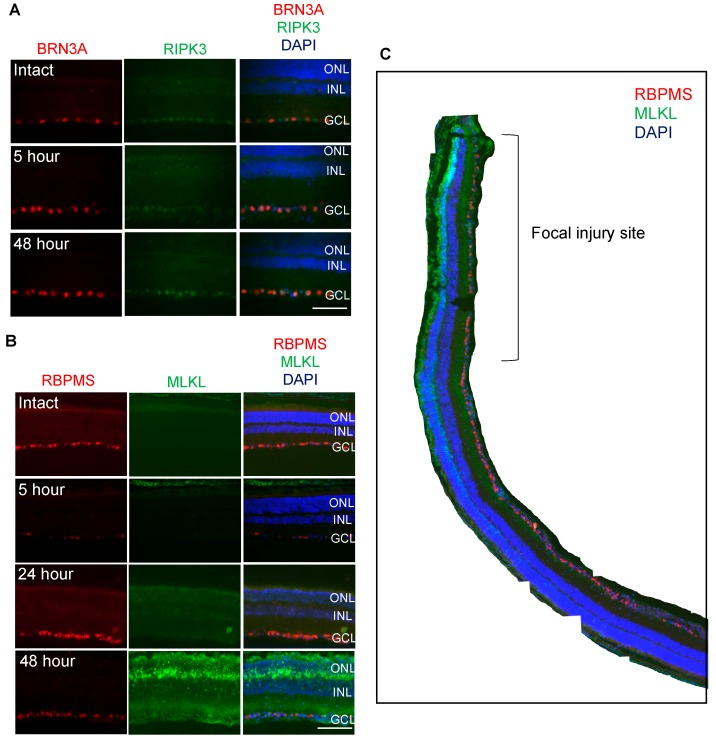
Immunofluorescent staining showed that receptor interacting protein kinase 3 (RIPK3) expression was no different at the focal impact site 48 h after blunt ocular injury compared with control eyes but Mixed Lineage Kinase Domain like Pseudokinase (MLKL) expression was higher. Images are taken from the same region of the injury site (representative of *n* = 4). (**A**) RIPK3 immunostaining was no different in Brain-specific homeobox/POU domain protein 3A (BRN3A)^+^ retinal ganglion cells (RGC) in the ganglion cell layer (GCL) at 5, 24 and 48 h after blunt ocular injury. (**B**) MLKL protein expression was low in intact eyes and 5 and 24 h after injury, but higher 48 h after injury and localized to the outer plexiform layer (OPL) as well as some outer nuclear layer (ONL) cells, inner nuclear layer (INL) cells and retinal binding protein with multiple splicing (RBPMS)^+^ RGC in the GCL. (**C**) Composite image of a retina 48 h after blunt injury. Immunostaining shows MLKL expression is localized specifically to the blunt ocular injury site. Nuclei were counterstained with DAPI (blue). Scale bar in Figure 3A represents 20 μm and Figure 3B represents 50 μm. Images are representative of *n* = four animals.

We then used immunofluorescent staining to determine the localization of RIPK3 and MLKL in the retina. RIPK3 showed co-localization with the RGC-specific marker, BRN3A and its staining intensity and localization appeared to be unchanged after blunt ocular trauma when compared to intact eyes (Figure 3A). MLKL immunofluorescence was elevated in cells at the focal impact site at 48 h post injury (Figure 3B), but not at 5 (Figure 3B) or 24 h, as shown in a composite image of the entire retina, with elevated expression mainly in the photoreceptor inner segments, outer plexiform layer (OPL) as well as some ONL cells, inner nuclear layer (INL) cells and RBPMS^+^ RGC (Figure 3C).

### 3.3. Nec-1s is Neuroprotective In Vitro

**Figure 4:**
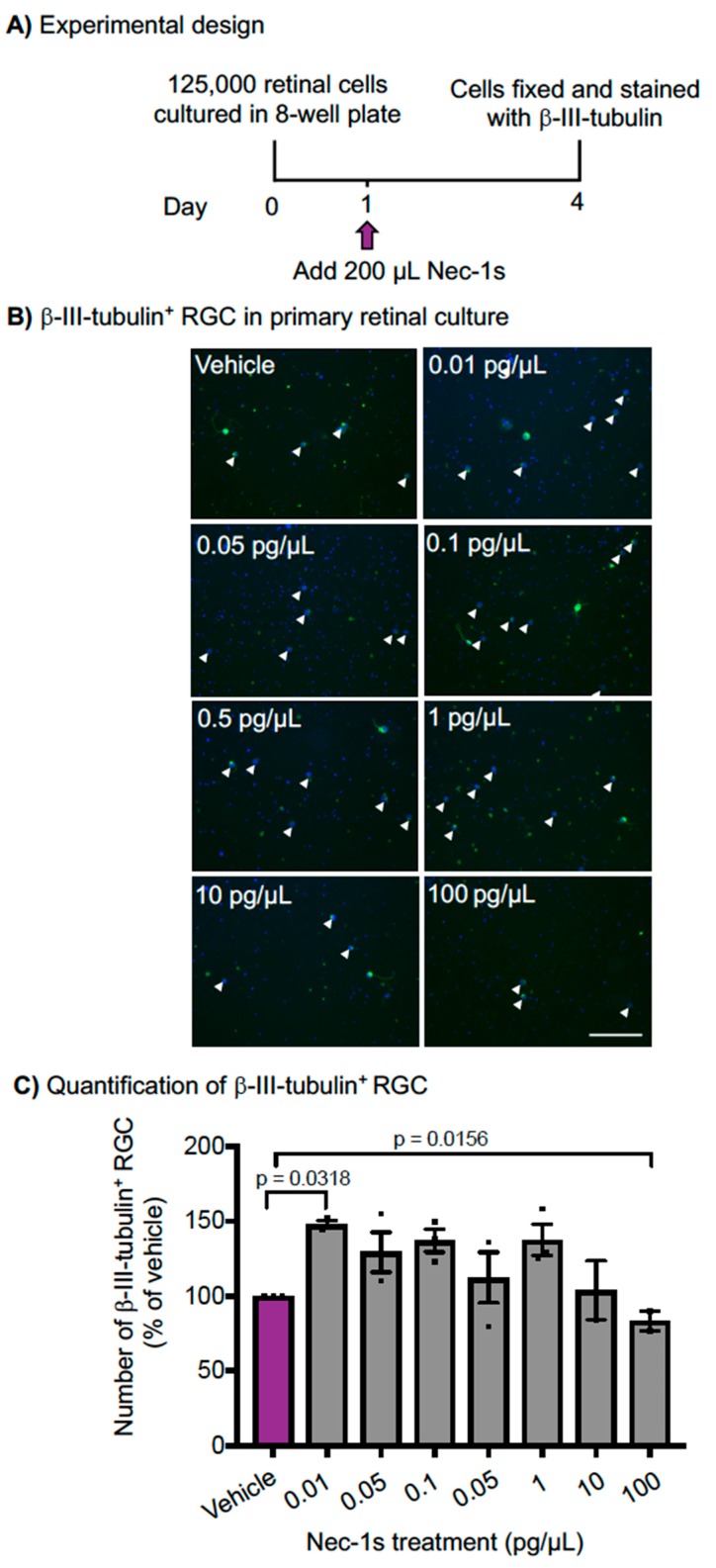
The number of β-III-Tubulin^+^ retinal ganglion cells (RGC) was higher after Necrostatin-1s (Nec-1s) treatment compared to vehicle in primary rat mixed retinal cultures. Treatment with varying doses of Nec-1s were performed in duplicate and performed on three independent occasions. (**A**) The experimental design of retinal culture experiments with Nec-1s treatment. Cells were plated at a 125,000 cell density in 300 μL supplemented media, after 1 day 200 μL of supplemented media containing Nec-1s was added, to make final concentrations of 0.01 to 100 pg/μL Nec-1s. (**B**) Representative images of β-III-Tubulin^+^ RGC (white arrow heads) treated with 100 to 0.01 pg/μL of Nec-1s. (**C**) The number of β-III-Tubulin^+^ RGC were quantified by taking 45 images per well, and displayed as mean percentages of vehicle controls. Nec-1s provided significant protection of β-III-Tubulin^+^ RGC compared to vehicle control treatment (PBS, 10% DMSO, 0.9% β-methyl-cyclodextrin). In Figure 4B, the scale bar represents 100 μm. Values are mean ± SEM.

To determine the contribution of necroptosis to RGC death in vitro, we used a specific, stable inhibitor of RIPK1 to inhibit necroptosis and measured RGC death in adult primary mixed retinal cultures (Figure 4A). Representative βIII-tubulin^+^ immunostained retinal cultures are shown in Figure 4B, with RGC highlighted with a white arrow. Retinal cultures were treated with a concentration of 100 to 0.01 pg/µL of Nec-1s and showed significant neuroprotection of βIII-tubulin^+^ RGC (*p* < 0.05, ANOVA; Figure 4B,C). The statistical effect of treatment was driven by 0.01 pg/µL Nec-1s, which caused a ~1.5-fold increase in βIII-tubulin^+^ RGC (*p* < 0.05, ANOVA, post-hoc Tukey with multiple comparisons *p* = 0.03; Figure 4C). At high concentrations Nec-1s (10–100 pg/µL), the number of βIII-tubulin^+^ RGC was similar to vehicle-treated cultures. These results demonstrate that Nec-1s is significantly neuroprotective to RGC in culture.

### 3.4. Nec-1s Preserved BRN3A^+^ and RBPMS^+^ RGC at the Center of the Blunt Injury Site Compared to Vehicle Treated Eyes but did not Preserve Photoreceptors

**Figure 5:**
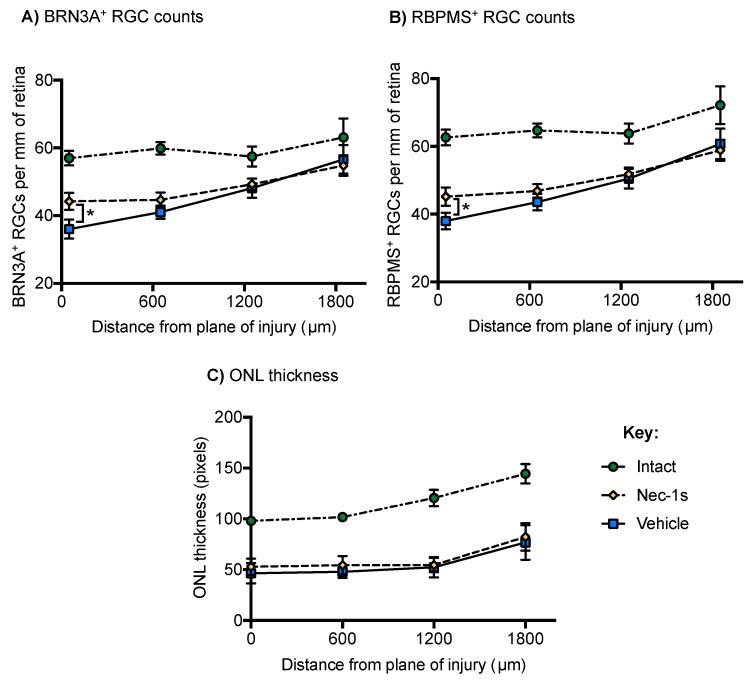
Necrostatin-1s (Nec-1s) treatment is protective of Brain-specific homeobox/POU domain protein 3A (BRN3A) ^+^ and retinal binding protein with multiple splicing (RBPMS)^+^ retinal ganglion cells (RGC) at the center of the impact site but does not preserve outer nuclear layer (ONL) thickness. (**A**,**B**) The number of BRN3A^+^ and RBPMS^+^ RGC were counted across entire retinal sections and reported as the number of RGC per mm of retina. Animals received bilateral blunt ocular injury with unilateral Nec-1s treatment and contralateral vehicle control. After unilateral intravitreal injection of Nec-1s, the number of BRN3A^+^ and RBPMS^+^ RGC was higher compared to vehicle control-treated eyes, across the retina with respect to retinal position (generalized linear models; BRN3A *p* < 0.01, RBPMS *p* < 0.05), likely driven by larger numbers of RGC detected at the center of the injury site. In the distant periphery (1200 μm, 1800 μm), there was less retinal degeneration overall and no significant effect of Nec-1s treatment on the number of BRN3A^+^ or RBPMS^+^ RGC compared to vehicle. (**C**) ONL thickness was reduced after blunt ocular injury and Nec-1s treatment did not preserve ONL thickness compared to vehicle control (generalized linear models; *p* = 0.313). Error bars represent mean ± SEM. *n* = 8 per group (intact = eight animals, *n* = eight bilateral blunt injured with unilateral Nec-1s intravitreal injection and contralateral vehicle intravitreal injection).

We then determined the contribution of necroptosis to RGC death after blunt ocular trauma in vivo. Bilateral blunt ocular injury was performed with unilateral intravitreal injection of Nec-1s and contralateral vehicle injection (n = eight eyes per condition, eight/ animals). The number of BRN3A^+^ (Figure 5A) and RBPMS^+^ (Figure 5B) RGC were quantified at varying distances from the center of the impact site (0, 600, 1200 and 1800 µm).

Nec-1s treated eyes had a greater number of BRN3A^+^ and RBPMS^+^ RGC at the center of the impact site compared to vehicle controls, with an effect that varied by distance from the impact site (GLM for BRN3A [*p* < 0.001] and RBPMS [*p* = 0.04] counts), with little effect of Nec-1s on the number of BRN3A^+^ and RBPMS^+^ RGC at distances more peripheral from the impact site (600, 1200 and 1800 µm; Figure 5A,B; model output in Table 2A,B). At the center of the impact site, there were 72.05% of RBPMS^+^ RGC in Nec-1s treated eyes, compared to 60.56% in vehicle treated eyes, and 77.64% BRN3A^+^ stained RGC in Nec-1s treated eyes, compared to 63.27% in vehicle treated control eyes.

Photoreceptor survival was assessed by ONL thickness measurements on H&E stained retinal sections at 600, 1200 and 1800 µm either side of the impact site (Figure 5C), as previously described [33,34]. There was no significant effect on ONL thickness in Nec-1s injected eyes compared to vehicle control, with no significant variation by distance from impact site (generalized estimating equations *p* = 0.313; model output in Table 2C). The normalized ONL thickness at 0, 600, 1200 and 1800 µm from impact site were 53.92%, 53.45%, 45.19% and 57.02% of intact retinae in Nec-1s treated eyes, compared to 47.36%, 47.09%, 43.4% and 53.30% in vehicle treated control eyes. Collectively, these results suggest that inhibition of the necroptotic pathway specifically protects RGC after blunt ocular trauma, but not photoreceptors.

### 3.5. Nec-1s Treatment did not Preserve ERG Amplitudes After Blunt Ocular Injury

**Figure 6:**
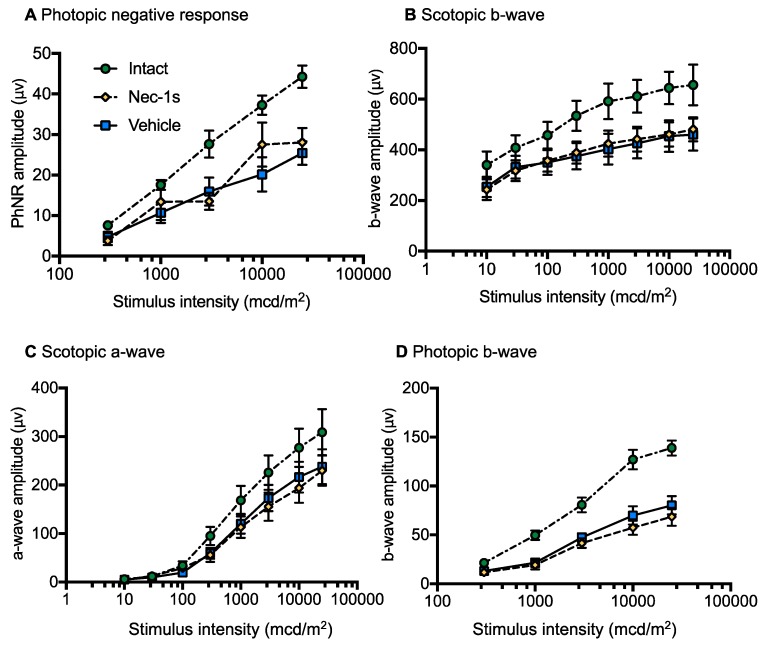
There were no differences in electroretinogram (ERG) amplitudes in Necrostatin-1s (Nec-1s) treated eyes compared to vehicle treated eyes after blunt ocular injury. (**A**) Photopic negative response (PhNR) amplitudes were reduced after blunt ocular injury and PhNR amplitudes were not increased by Nec-1s treatment compared to vehicle control. (**B**) Scotopic b-wave; (**C**) scotopic a wave and; (**D**) photopic b-wave amplitudes were reduced after blunt ocular injury and there was no improvement with Nec-1s treatment compared to vehicle control. Error bars represent mean ± SEM. *n* = eight per group (intact = eight animals, *n* = eight bilateral blunt injured with unilateral Nec-1s intravitreal injection and contralateral vehicle intravitreal injection).

We then determined if RGC neuroprotection close to the injury site led to preservation of RGC function. Scotopic and photopic ERG recordings were performed at 14 dpi and compared to between Nec-1s and vehicle-treated eyes (*n* = eight animals) to assess retinal function. Intact animals receiving no injections were also analyzed (*n* = eight animals). There were reductions in the PhNR (Figure 6A), photopic b-wave (Figure 6B), scotopic a-wave (Figure 6C) and scotopic b-wave (Figure 6D) amplitudes after blunt ocular injury compared to intact animals, but there was no improvement in amplitudes in Nec-1s treatment eyes compared to vehicle treated eyes (Figure 6A–D). These results demonstrate that, although more RGC were neuroprotected after treatment with Nec-1s, compared to intact eyes their function was still impaired.

## 4. Discussion

We have shown elevated retinal levels of necroptosis pathway proteins, including MLKL in RGC and other retinal cells. We further show that treatment with a necroptosis inhibitor, Nec-1s, was neuroprotective to RGC in vitro and prevented RGC loss at the center of the impact site in a rat model of closed-globe blunt ocular injury in vivo.

MLKL protein expression was higher after blunt ocular injury compared with intact retina, but we did not detect changes in RIPK1 or RIPK3 protein levels. RIPK3 was localized to BRN3A^+^ RGC, although there were no significant changes in protein levels up to 48 h after injury, suggesting that RIPK3 levels were not altered by injury. The lack of RIPK3 upregulation, despite other features of necroptosis being present, may relate to the post-translational nature of RIPK activation or the existence of RIPK3-independent pathways [44]. MLKL immunostaining, however, was localized to cells in the OPL, INL, ONL and RGC at 48 h after blunt injury at the center of the impact site, suggesting that multiple retinal cell types, including photoreceptors, may have upregulated MLKL expression, and hence activated necroptotic signaling pathways. MLKL had the greatest increase in protein expression at 24 h in Western blotting, but no immunostaining until 48 h, which may be explained by differences in detection technique. Western blotting runs denatured proteins on an SDS-gel after separating protein complexes (e.g., necrosome) and opening protein structure, while PFA fixation used for immunostaining provides a snapshot of proteins in a fixed state and with intact protein complexes. During necroptosis, MLKL complexes with RIP1 and RIP3 in complex IIb, leading to phosphorylation of the pseudokinase domain (adjacent to the “brace” region recognized by our antibody [45]), which induces its oligomerization and translocation to the plasma membrane. The epitope recognized by our antibody may therefore have been blocked in complex IIb and more readily recognized after phosphorylation, oligomerization and plasma membrane translocation, meaning that we detected increased MLKL protein levels and its epitope earlier in the course of MLKL production and activity by Western blotting because of the open structure of the protein in this technique, but only later by immunohistochemistry after conformational and binding changes. Necroptotic photoreceptor death occurs in in vivo models of retinal detachment and age-related macular degeneration [46,47], suggesting that necroptosis may also drive photoreceptor degeneration after blunt ocular injury.

The phosphorylation of MLKL at Ser345 is catalyzed by RIPK1 and promotes MLKL translocation and accumulation at the plasma membrane, inducing membrane permeabilisation and cell lysis [22,23], although the association of RIPK3 and MLKL in the necrosome is not dependent on Ser345 phosphorylation [22]. We have shown MLKL expression is upregulated 24–48 h after injury. Immunofluorescent staining in the OPL suggests that MLKL is present in the connections between the photoreceptors and INL cells.

For necroptotic signaling to be activated and induce cell death, apoptotic caspase-8 must be inhibited either through endogenous or pharmacological inhibitors or remain uncleaved. This allows for the formation of complex IIb and the association of RIPK1, RIPK3 and MLKL [20]. Blanch et al., showed that caspase-8 remained uncleaved up to 48 h after blunt ocular injury [34], with no retinal caspase-8 immunostaining and unbiased caspase-capture experiments using b-VAD-fmk, confirming that there was no caspase-8 activation since no cleaved caspase-8 was isolated, indicating that caspase-8-independent cell death signaling drives retinal cell death after blunt ocular injury [34].

RIPK1 is a key mediator of necroptotic cell death, but not exclusively, and also has roles in apoptotic and inflammatory pathways [44]. Similarly, MLKL can have roles in death-receptor-induced apoptosis [48] and inflammasome activation [49,50]. We detected no changes in RIPK1 protein expression after blunt ocular injury up to 48 h, but we do show increased MLKL specifically at the focal impact site. Blanch et al., have shown no retinal activation of apoptotic caspase-8, implying that the extrinsic apoptotic pathway is not occurring in the retina after injury. This is consistent with our previous studies demonstrating that intrinsic caspase-9 dependent and the non-canonical caspase-2 dependent pathways drive a proportion of retinal death in the blunt ocular injury model [33,34,51]. The necroptosis inhibitor, Nec-1s, is a synthetic allosteric inhibitor of RIPK1 and inhibits necroptotic cell death through preventing the transition from Complex IIa to Complex IIb, by inhibiting the interaction of RIPK1 and RIPK3 [21,52,53].

After ON injury, RGC death begins within 5 days and there is >90% RGC loss at 14 dpi [16]. Adult RGC begin to die in retinal cultures due to the disrupted RGC axonal connections. Previous studies have used the same timeframe for retinal cultures and shown effective results from a variety of treatments [42,43]. There was a significant neuroprotective effect in culture after Nec-1s treatment compared to vehicle control, suggesting that necroptosis signaling mediates the death of a proportion of RGC. At low doses of Nec-1s (0.01 pg/µL), there was significant improvement in RGC numbers, but at higher doses (100 pg/µL) there was no RGC neuroprotection, indicating that low concentrations of Nec-1s may be more effective in vitro. Neuroprotection in vitro in primary retinal cell cultures indicates that necroptotic cell death pathways can mediate degeneration of injured RGC but does not take into account drug clearance and metabolism and immune response. However, as necroptosis can be linked to inflammation [54,55], primary retinal cultures are, therefore, not fully representative of in vivo signaling pathways, but provide a screening platform to identify RGC neuroprotective agents [35,41,42,56,57]. At lower concentrations of Nec-1s, necroptosis might be reduced rather than fully inhibited, which might be more beneficial. Cell death pathways are interlinked and, if one pathway is reduced, another might be activated, for example, inhibition of caspase-8 dependent apoptosis promotes necroptosis [58]. DMSO is used to solubilize the compounds but can be neurotoxic to RGC at high concentrations. For example, in vitro, concentrations of >10% DMSO induce RGC apoptosis and plasma membrane pore formation [59,60]. We reconstituted Nec-1s in 10% DMSO and 0.9% methyl-β-cyclodextrin to aid with solubility. Despite the potential of DMSO to cause RGC death at higher concentrations, we have shown consistent retinal pathology across studies, suggesting DMSO had a minimal effect in our study [15,33,34].

**Figure 7:**
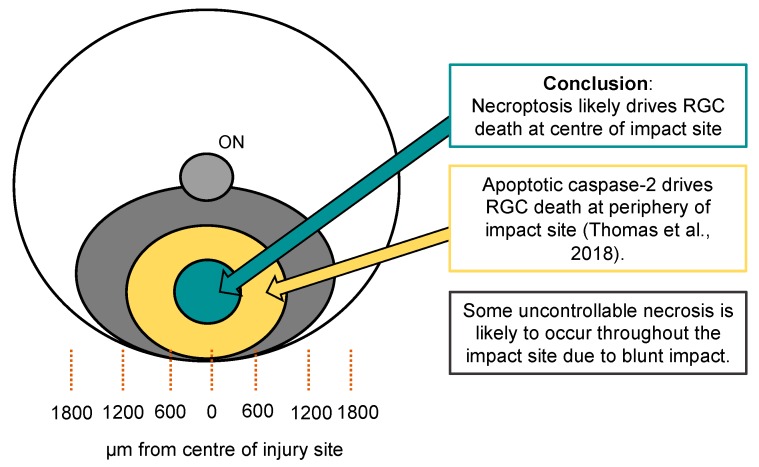
Diagram of the back of the retina, showing the focal impact site from the blunt ocular injury model. The green central zone represents retinal ganglion cells (RGC) death driven by necroptotic signaling pathways and there was a greater number of retinal binding protein with multiple splicing (RBPMS)^+^ and Brain-specific homeobox/POU domain protein 3A (BRN3A)^+^ RGC in this area compared to untreated control eyes. Our previous publication showed caspase-2 drives RGC death in the immediate periphery of the impact site. Uncontrolled necrosis is likely to occur throughout the impact site, due to the impact of the blunt projectile.

Our in vivo rat blunt ocular injury model induces retinal cell death in a focal area, causing photoreceptor death and RGC degeneration [15,33,34]. We previously proposed that RGC death is driven by caspase-2 in the immediate periphery (600 µm) to the center of the impact site, and now propose that necroptosis drives RGC death at the center of the impact site. In our previous publication, small interfering RNA (siRNA)-mediated caspase-2 knockdown protected a proportion of RGC and preserved electroretinographic RGC function [33]. In the immediate periphery of the impact site, siRNA-mediated caspase-2 knock preserved 85% of BRN3A^+^ RGC compared to 67% in siEGFP-treated blunt injured eyes, suggesting that 18% of RGC in this region degenerated by a process of caspase-2 dependent apoptosis. siCASP2 had no detectable effect on RGC survival at the impact site center [33], despite preserving >98% RGC in the ONC model [61], suggesting that other cell death pathways mediate RGC degeneration after blunt ocular injury at the center of the impact site. Electron microscopic evidence suggests that injury is most severe at the center of the impact site, with more extensive cell death and necrotic, rather than apoptotic morphology [15]. We propose that RGC death in the center of the impact site, is mediated by necroptotic mechanisms, evidenced by elevated MLKL and increased numbers of RGC compared to vehicle control in this region. After Nec-1s injection, 78% of BRN3A^+^ RGC were preserved in the center of the impact site, compared to 63% after control vehicle injection, suggesting that 15% of RGC in this region undergo necroptotic cell death. We did not observe any evidence of necroptotic death outside the center of the impact site, and in our previous paper we only observed evidence of caspase 2-dependent RGC death in the region peripheral to the impact site and none at the impact site center. This implies that caspase-2 and necroptotic death pathways occur simultaneously in the same cell type but in different regions of the impact site, probably related to the severity of injury at the different locations (Figure 7). However, Nec-1s (or siCASP2 [33]) treatment did not preserve the number of RGC to levels found in intact retina, suggesting that whilst some RGC degeneration was mediated by necroptotic signaling pathways, a proportion of RGC death at the center of the impact site occurs through other pathways, such as uncontrolled necrosis.

In line with our findings, RGC are also protected by the inhibition of necroptosis in other models of RGC death, including glaucoma [62] and retinal ischemia-reperfusion injury [63], and other models of neuronal disease and degeneration, including amyotrophic lateral sclerosis [64,65,66], Alzheimer’s disease [26], Niemann–Pick disease [67], traumatic brain injury [28] and spinal cord injury [27]. The abundance of studies across multiple disease indicates the importance of necroptotic signaling in driving neuronal cell death.

Caspase-2 can negatively regulate necroptosis, and downregulation of caspase-2 using shRNA or CRISPR/Cas9 system enhances phosphorylation of RIPK1 and MLKL [68]. In our studies, siCASP2-mediated knock down improved RGC survival, but the number of preserved RGC might have been underrepresented; if caspase-2 suppression caused necroptosis activation this could have caused death in some RGC, limiting the neuroprotective efficacy of siCASP2. The converse is unlikely to be true, as we are not aware of any data suggesting modulation of caspase-dependent death by necroptotic mechanisms. Furthermore, apoptotic and necroptosis death mechanisms can occur simultaneously in the same cell type [69], consistent with the observations that both caspase-2 and necroptotic-dependent death occur simultaneously in our model.

RGC were quantified by counting BRN3A^+^ (RGC-specific transcription factor with nuclear immunostaining) and RBPMS^+^ (cytoplasmic marker) immunostained cells in the GCL [37,70]. BRN3A only stains a subset of RGC and its expression can be reduced after RGC injury [38,71,72]. RBPMS immunolabels a greater proportion of RGC subpopulations [39,40], but has yet to be validated as a reliable RGC marker [37]. Nonetheless, we demonstrated significant RGC neuroprotection using both BRN3A and RBPMS markers after Nec-1s treatment following blunt ocular injury, with similar cell counts by both methods.

Photoreceptor cell bodies populate the ONL; therefore, ONL thickness can be used to assess photoreceptor survival [15,34]. In the rd10 mouse retinitis pigmentosa model, inhibition of RIPK1 using Nec-1 or *ripk3* gene knockout preserved cone photoreceptors and function [73], and photoreceptor necroptosis also occurs in models of age-related macular degeneration [74] and a model of retinal detachment [46]. As an alternative to intravitreal injections, Nec-1s was delivered through subcutaneous delivery and rescued ONL thinning in a mouse P23H rhodopsin model of retinal degeneration [75], suggesting that Nec-1s can have an effect in the retina through systemic and non-local delivery. In the blunt ocular injury model, intrinsic apoptotic pathway member caspase-9 mediates degeneration of some photoreceptors in the far periphery of the impact site and caspase-9 inhibition using a dominant negative mutant protein preserved ERG amplitudes [34]. Despite the immunolocalization of MLKL to the ONL, OPL and photoreceptor inner segments, treatment with Nec-1s did not preserve of ONL thickness compared to vehicle, which could be explained by (1) necroptosis not mediating photoreceptor death after blunt injury or; (2) necroptosis inhibition promoting activation of an alternative cell death signaling pathway.

Scotopic (dark-adapted) and photopic (light-adapted) ERG amplitudes were measured to assess retinal function. The PhNR is a common measure of RGC function; it is reduced in experimental and human glaucoma [76] and correlates with reduced RGC numbers after ON transection [77]. Treatment with Nec-1s had no effect on scotopic or phototopic a- or b- wave amplitudes and did not affect PhNR amplitudes, suggesting that retinal function, including photoreceptor and RGC function, was not improved by Nec-1s treatment. The lack of functional restitution suggests that some RGC are structurally protected by Nec-1s but may be non-functional, thus detecting no differences in PhNR amplitude. It is also possible that the focal nature of the retinal injury and the specific neuroprotection of RGC at the center of the impact site may mean that too few RGC are preserved and functional enough to be detectable by full-field ERG. Focal changes in retinal function may be more sensitively detected using focal or pattern ERG in future investigations. The complete absence of any trend towards improved function on ERG suggests that this is not the case, and a reduction in PhNR amplitude with blunt injury despite the focal impact also discourages this explanation.

## 5. Conclusions

In conclusion, we have demonstrated that RGC death signaling pathways are active in a region-specific manner after blunt ocular injury, with necroptotic-dependent mechanisms likely driving a reduction in RGC numbers at the center of the impact site. The inhibition of necroptosis using pharmacological inhibitor, Nec-1s, preserved the number of RBPMS^+^ and BRN3A^+^ RGC compared to vehicle. This suggests that Nec-1s might protect a proportion of RGC, but, as there was no increase in ERG amplitudes, Nec-1s did not provide any corresponding improvement in retinal function. We have previously shown that caspase-2-dependent RGC death occurred peripheral to the impact site, which, collectively with this study, suggests that multiple cell death signaling pathways can occur in parallel in the same tissue and in the same cell type, in a single injury model.

## Figures and Tables

**Table 1:** List of primary and secondary antibodies used for western blot (WB) and immunohistochemistry (IHC).

**Antigen (Origin)**	**Dilutions**	**Company**	**Catalogue Number**
RIPK1 (mouse)	1:200 (WB)	BD Pharmigen	551041
RIPK3 (B2) (mouse)	1:200 (IHC)	Santa Cruz	SC374639
RIPK3 (rabbit)	1:1000 (WB)	Abcam	AB56164
MLKL (rat)	1:5000 (WB), 1:1000 (IHC)	Millipore	MABC604
BRN3A (C-20) (goat)	1:200 (IHC)	Santa Cruz	SC-31984
RBPMS (rabbit)	1:400 (IHC)	Millipore	ABN1362
β-actin (mouse)	1: 10,000 (WB)	Sigma	A5441
βIII-tubulin (mouse)	1:200 (ICC)	Sigma	T8660
**Secondary Antibodies (Origin)**	**Dilutions**	**Company**	**Catalogue Number**
Anti-goat Alexa Fluor 594 (goat)	1:400 (IHC)	Invitrogen	A11058
Anti-rabbit Alexa Fluor 488 (donkey)	1:400 (IHC)	Invitrogen	A21206
Anti-rabbit Alexa Fluor 594 (donkey)	1:400 (IHC)	Invitrogen	A21207
Anti-rat Alexa Fluor 488 (goat)	0.736111	Invitrogen	A11006
Anti-rabbit HRP-linked (goat)	1:1000 (WB)	Cell Signalling Technologies	7074S
Anti-rat HRP-linked (goat)	1:5000 (WB)	Cell Signalling Technologies	7077S
Anti-mouse HRP-linked (horse)	1:1000 (WB)	Cell Signalling Technologies	7076S

**Table 2:** Mean number of Brain-specific homeobox/POU domain protein 3A (BRN3A)^+^ and retinal binding protein with multiple splicing (RBPMS)^+^ retinal ganglion cells (RGC) and outer nuclear layer (ONL) thickness after blunt ocular injury and treatment with Necrostatin-1s (Nec-1s) injections. (**A**,**B**) BRN3A^+^ and RBPMS^+^ RGC counts in intact animals and after blunt ocular injury treated with Nec- 1s or vehicle intravitreal injections. The values represent the mean number of BRN3A^+^ or RBPMS^+^ RGC per mm of retina (95% confidence intervals). (**C**) ONL thickness in intact animals and after blunt ocular injury treated with Nec-1s or vehicle intravitreal injections. Values are mean pixels of ONL (95% confidence intervals).

**A**	**Mean Number of BRN3A^+^ RGC per mm of Retina (95% CI)**			
Treatment	0 µm	600 µm	1200 µm	1800 µm
Intact	56.95	59.87	57.48	63.10
	(52.7–61.2)	(56.2–63.5)	(51.7–63.3)	(42.2–74.0)
Blunt + Nec-1s	44.2	44.7	49.3	54.7
	(39.1–50.0)	(35.9–55.6)	(36.9–65.9)	(39.5–75.9)
Blunt + vehicle	36.0	41.0	48.1	56.8
	(27.0–48.2)	(24.8–67.8)	(25.4–91.3)	(27.4–118)
*p* value for comparison	*p* = 0.017	n/a	n/a	n/a
**B**	**Mean Number of RBPMS^+^ RGC per mm of Retina (95% CI)**			
Treatment	0 µm	600 µm	1200 µm	1800 µm
Intact	62.68	64.70	63.84	72.16
	(58.2–67.2)	(60.7–68.7)	(58.0–69.7)	(61.3–83.6)
Blunt + Nec-1s	45.2	46.9	51.8	58.9
	(40.4–50.5)0	(37.9–57.9)	(39.6–67.8)	(44.7–77.6)
Blunt + vehicle	38.0	43.6	50.5	60.9
	(29.3–49.1)	(27.4–69.3)	(29.0–87.9)	(33.0–112.6)
*p* value for comparison	*p* = 0.02	n/a	n/a	n/a
**C**	**Mean Number of Pixels of ONL Thickness (95% CI)**			
Treatment	0 µm	600 µm	1200 µm	1800 µm
Intact	98.12	101.72	120.66	144.34
	(94.4–101.9)	(94.1–109.4)	(104.9–136.4)	(125.6–163.1)
Blunt + Nec-1s	52.91	54.36	54.53	82.31
	(45.1–60.7)	(45.5–63.3)	(47.6–61.5)	(76.8–87.8)
Blunt + vehicle	46.47	47.90	52.38	76.79
	(36.6–56.3)	(42.0–53.7)	(42.5–62.3)	(69.0–93.5)
*p* value for comparison	*p* = 0.229	*p* = 0.292	*p* = 0.625	*p* = 0.143

## Abbreviations

ANOVA	Analysis of Variance
BRN3A	Brain-Specific Homeobox/POU Domain Protein 3A
BSA	Bovine Serum Albumin
DAPI	4′,6’-Diamidino-2-Phenylindole
DMSO	Dimethyl Sulfoxide
Dpi	Days Post Injury
ERG	Electroretinogram
GCL	Ganglion Cell Layer
GLM	Generalized Linear Models
H&E	Hematoxylin and Eosin
IDO	Indoleamine-2,3-Dioxygenase
IHC	Immunohistochemistry
INL	Inner Nuclear Layer
MLKL	Mixed Lineage Kinase Domain Like Pseudokinase
Nec-1	Necrostatin-1
Nec-1s	Necrostatin-1s
OCTc	Optimal Cutting Temperature Compound
ONC	Optic Nerve Crush
ON	Optic Nerve
ONH	Optic Nerve Head
ONL	Outer Nuclear Layer
OPL	Outer Plexiform Layer
PBS	Phosphate Buffered Saline
PFA	Paraformaldehyde
PVDF	Polyvinylidene Fluoride
RBPMS	Retinal Binding Protein with Multiple Splicing
RGC	Retinal Ganglion Cells
RT	Room Temperature
RIPK1	Receptor Interacting Kinase 1
RIPK3	Receptor Interacting Kinase 3
SDS	Sodium Dodecyl Sulphate
SEM	Standard Error of the Mean
siRNA	Small Interfering RNA
TON	Traumatic Optic Neuropathy
TNF	Tumor Necrosis Factor
WB	Western Blot
