# Addendum: Thomas et al. Retinal Ganglion Cells Die by Necroptotic Mechanisms in a Site-Specific Manner in a Rat Blunt Ocular Injury Model. *Cells* 2019, *8*, 1517

**DOI:** 10.3390/cells10050974

**Published:** 2021-04-21

**Authors:** Chloe N. Thomas, Adam M. Thompson, Zubair Ahmed, Richard J. Blanch

**Affiliations:** 1 Neuroscience and Ophthalmology, Institute of Inflammation and Ageing, University of Birmingham, Birmingham B15 2TT, UK; c.thomas.4@bham.ac.uk (C.N.T.); a.thompson@exeter.ac.uk (A.M.T.); 2 School of Biomedical Sciences, Institute of Clinical Sciences, College of Medical and Dental Sciences, University of Birmingham, Birmingham B15 2TT, UK; 3 Academic Department of Military Surgery and Trauma, Royal Centre for Defence Medicine, Birmingham B15 2TT, UK; 4 Department of Ophthalmology, University Hospitals Birmingham NHS Foundation Trust, Birmingham B15 2TT, UK

The authors would like to make an addendum to their published paper [1]. 

Details of pre- and post-procedural analgesia were missing in the original version of the article in Section 2.2.

The authors wish to add that “pre- and post-procedural analgesia was administered to all animals undergoing procedures”.

The change does not affect the scientific results. 

The rest of the manuscript does not need to be changed. The authors would like to apologize for any inconvenience caused.
